# Reducing Salinity by Flooding an Extremely Alkaline and Saline Soil Changes the Bacterial Community but Its Effect on the Archaeal Community Is Limited

**DOI:** 10.3389/fmicb.2017.00466

**Published:** 2017-03-27

**Authors:** Arit S. de León-Lorenzana, Laura Delgado-Balbuena, Cristina Domínguez-Mendoza, Yendi E. Navarro-Noya, Marco Luna-Guido, Luc Dendooven

**Affiliations:** ^1^Soil Ecology Lab, Centro de Investigación y de Estudios Avanzados del IPN, CINVESTAVMexico, Mexico; ^2^Centro Tlaxcala de Biología de la Conducta, Cátedras CONACYT, Universidad Autónoma de TlaxcalaTlaxcala, Mexico

**Keywords:** extreme salinity, alkalinity, soil characteristics, bacterial and archaeal community structure, microbial successions

## Abstract

Regular flooding of the soil to reduce salinity will change soil characteristics, but also the microbial community structure. Soil of the former lake Texcoco with electrolytic conductivity (EC) 157.4 dS m-1 and pH 10.3 was flooded monthly in the laboratory under controlled conditions for 10 months while soil characteristics were determined and the archaeal and bacterial community structure monitored by means of 454 pyrosequencing of the 16S rRNA gene. The EC of the soil dropped from 157.8 to 1.7 dS m-1 and the clay content decreased from 430 to 270 g kg-1 after ten floodings, but the pH (10.3) did not change significantly over time. Flooding the soil had a limited effect on the archaeal community structure and only the relative abundance of Haloferax-like 16S rRNA phylotypes changed significantly. Differences in archaeal population structure were more defined by the initial physicochemical properties of the soil sample than by a reduction in salinity. Flooding, however, had a stronger effect on bacterial community structure than on the archaeal community structure. A wide range of bacterial taxa was affected significantly by changes in the soil characteristics, i.e., four phyla, nine classes, 17 orders, and 28 families. The most marked change occurred after only one flooding characterized by a sharp decrease in the relative abundance of bacterial groups belonging to the Gammaproteobacteria, e.g., Halomonadaceae (Oceanospirillales), Pseudomonadaceae, and Xanthomonadaceae and an increase in that of the [Rhodothermales] (Bacteroidetes), Nitriliruptorales (Actinobacteria), and unassigned Bacteria. It was found that flooding the soil sharply reduced the EC, but also the soil clay content. Flooding the soil had a limited effect on the archaeal community structure, but altered the bacterial community structure significantly.

## Introduction

The structure and composition of soil microbial communities are shaped by a hierarchy of abiotic and biotic characteristics that define which species and traits are present at a site (Dequiedt et al., 2011; Andrew et al., 2012). Soil characteristics and stochastic processes, such as disturbances, define temporal, and spatial heterogeneity of microbial community assembly (Lozupone and Knight, 2007; Fierer and Lennon, 2011). Measures of community composition and diversity are often used to infer the responses of community assembly to environmental gradients or other factors. These community indices provide strong evidence about which species occur in a given set of soil characteristics.

The soil of the former lake Texcoco is extremely saline and alkaline with pH often >10 and electrolytic conductivity (EC) > 100 dS m^−1^ (Dendooven et al., 2010). Vegetation is limited and in the most saline parts bare soil dominates while in other parts patches with *Distichlis spicata* L., a salt resistant grass of the family Poaceae (Gramineae), can be found. Bare soil was prone to wind erosion in the dry season causing dust storms in Mexico City aggravated by pathogens carried with the soil particles. “*Comisión Nacional de Agua*” (CNA, the Mexican National Water Commission) started a program to drain the saline soil and vegetate it in the 1970's. The most saline parts were flooded regularly with effluents from a nearby treatment plant strongly reducing salinity over the years (Luna-Guido et al., 2000). The program succeeded in creating nearly normal soil conditions (pH < 8.5 and EC < 5 dS m^−1^) in some parts, which allowed trees, such as *Tamarix* spp., and other Poaceae to growth.

Flooding the former lake bed had a strong effect on the microbial populations and different geochemical cycles, but especially on the dynamics of C and N. For instance, in the undrained soil, mineralization of applied organic material, i.e., glucose or maize, was low, but after >10 y of flooding, mineralization rates were nearly similar as in a non-saline soil. The bacterial and archaeal community structure changed substantially in soil flooded >10 y (Valenzuela-Encinas et al., 2008, 2009). Different experimental approaches have been applied to better understand the effect of flooding on the microbial community. Valenzuela-Encinas et al. (2008, 2012) first studied the archaeal communities using cloned sequences of the 16S rRNA gene in a pristine and drained soil. They also analyzed the bacterial communities in soil of the former lake Texcoco with different EC and pH following different periods of irrigation and drainage (Valenzuela-Encinas et al., 2009). Navarro-Noya et al. (2015) investigated and identified the archaeal diversity patterns in soil of the former lake Texcoco with different EC and pH. They concluded that the soil characteristics defined the archaeal community structure.

Studying soil samples from the former lake bed flooded and drained for different periods gave important insights into how the decreasing salinity and pH changed the geochemical cycles and the microbial community structure. However, not only salt content and pH were affected when the field was flooded, but other characteristics, such as vegetation, organic matter content, and particle size distribution, were different between the soils sampled. Some of these variations were due to flooding and drainage, but others due to the intrinsic variation of the former lake bed. Defining how salinity and alkalinity affected microbial populations was not always straightforward. The objective of this work was to determine the effect of a decreasing salt content on the archaeal and bacterial populations while other characteristics that might affect them were kept constant.

## Materials and methods

### Soil sampling site

The soil of the former lake bed is located in the valley of Mexico City (Mexico) at an altitude of 2, 240 masl with a mean annual temperature of 16°C and annual precipitation of 705 mm. Details of the soil characteristics and vegetation can be found in Luna-Guido et al. (2000). Soil was sampled haphazardly with a stony soil auger with a diameter of seven cm (Eijkelkamp, Giesbeek, Nl) by augering 30-times the 0–15 cm layer of three different plots with a size of *ca*. 400 m^2^. As such, approximately 30 kg soil was collected from each plot. The 30 samples taken in each plot were pooled, so that one soil sample was obtained from each plot. As such, a total of three different soil samples were obtained and taken to the laboratory. This field-based replication was maintained in the laboratory experiment. Sampling location data can be found in Supplementary Table S1.

### Preparation and incubation of the soil columns

The soil from each plot (*n* = 3) was passed separately through a 5 mm sieve, adjusted to 40% water holding capacity (WHC) and was pre-incubated separately in drums for 7 days at room temperature (22 ± 2°C). Each drum with a volume of 70 L contained 10 kg of soil and was closed airtight. The drums contained a beaker with 500 ml distilled H_2_O to avoid desiccation and a beaker with 1,000 mL 1 M NaOH solution to trap CO_2_ evolved. The drums were opened every day to avoid anaerobic conditions in the soil. It is clear, however, that anaerobic micro-sites cannot be excluded from the soil even when spreading out the soil and airing every day. Soil is generally pre-incubated (conditioned) so that the bacterial community can adapt to the new condition (i.e., changes in water content) and the organic material released upon sampling is mineralized (Franzluebbers and Arshad, 1997).

After 7 days, 8 sub-samples of 1.5 kg soil from each plot (*n* = 3) were packed separately into PVC columns with diameter 10.5 cm and length 30 cm with a mean bulk density of 0.95 g cm^−3^ (Dendooven et al., 2015). The columns were fitted at the bottom with thin plastic plates with holes drilled equally distributed over its surface. On top of it, a polyethylene filter disc (nominal pore size 0.5 μm) was placed and a layer of acid washed sand 0.16 kg to prevent loss of soil particles during leaching (Bellini et al., 1996).

**Figure 1:**
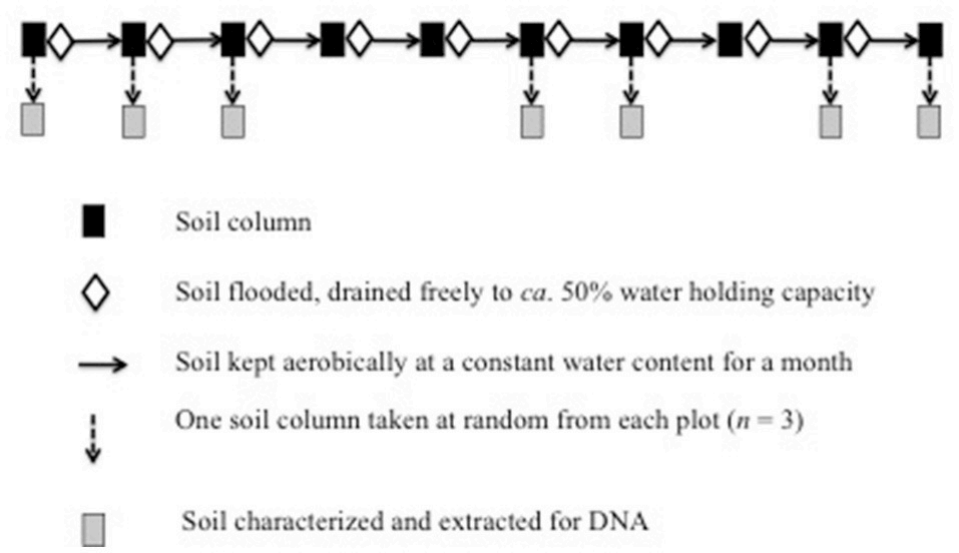
**Experimental design**.

The experimental design is given in Figure 1. One column from each plot (*n* = 3) were selected at random, the soil was removed, characterized and extracted for DNA. This soil never flooded was considered the control (Supplementary Table S2). The remaining columns were flooded with 3 L distilled water and drained freely until approximately 50% WHC. The top of the columns was fitted with parafilm to avoid drying of the soil, but to allow aeration. The soil was then conditioned aerobically and at constant water content (approximately 50% WHC) for 1 month. After 1 month, the soil was removed from the column, extracted for DNA and the WHC determined. The WHC of the soil was determined after flooding as the reduction in salt content might change its capacity to retain water. The soil was flooded again with 3 L distilled water and drained freely until approximately 50% WHC. This process of flooding the soil, draining the soil freely until *ca*. 50% WHC, covering the column with parafilm and conditioning the soil for a month was repeated monthly until the soil was flooded 10 times. In a previous experiment, it was found that flooding the soil monthly eight times was sufficient to decrease the EC < 5 dS m^−1^ (Dendooven et al., 2015). After 2, 3, 6, 7, 9, and 10 months, one column was selected at random from each plot (*n* = 3), the soil was removed, characterized, the WHC determined and extracted for DNA (Supplementary Table S2).

### Soil characterization

The pH was determined in 1:2.5 soil–H_2_O suspension using a calibrated Ultra Basic UB-10 pH/mV meter (Denver Instrument, NY, USA) with a glass electrode (#3007281 pH/ATC, ThermoFisher Scientific, Waltham, Massachusetts, USA). Electrolytic conductivity (EC) was measured with a portable microprocessor HI 933300 (HANNA Instruments, Woonsocket, Rhode Island, USA). The WHC was measured on soil samples water-saturated in a funnel, covered with an aluminum foil to avoid water evaporation and left to stand overnight to drain freely and was defined by differences of weight. The soil particle size distribution was determined by the hydrometer method as described by Gee and Bauder (1986).

### DNA isolation and PCR-amplification of bacterial and archaeal 16S rRNA genes

Sub-samples of 0.5 g soil were washed with 0.15 M sodium pyrophosphate and 0.15 M phosphate buffer pH 8 to remove the humic acids (Ceja-Navarro et al., 2010). Metagenomic DNA was extracted with three different techniques (Hoffman and Winston, 1987; Sambrook and Russell, 2001; Valenzuela-Encinas et al., 2008). Each technique was used to extract DNA from 0.5 g, then, the DNA from the three different techniques was pooled. As such, DNA was extracted from 1.5 g of each soil sample. The metagenomic DNA was used to amplify the region V1–V3 of the bacterial and archaeal 16S rRNA gene using the set of primers 8-F (5′-AGA GTT TGA TCI TGG CTC A-3′) and 556-R (5′-TGC CAG IAG CIG CGG TAA-3′) and primers 25F 5′–CYG GTT GAT CCT GCC RG–3′ (Dojka et al., 1998) and A571R 5′–GCT ACG GNY SCT TTA RGC–3′ (Baker et al., 2003) for Bacteria and Archaea, respectively. Primers contain a 10 bp barcode and the Roche 454 pyrosequencing adaptors Lib-L.

The PCR mixture (25 μl) contained 1 × reaction buffer, 10 mmol L^−1^ of each of the four deoxynucleoside triphosphates, 10 pmol L^−1^ with each of the primers, 1.0 U Taq polymerase (Thermo Scientific), and 20 ng metagenomic DNA as template. The following thermal cycling was used for the amplification of bacteria: initial denaturation at 95°C for 10 min, 25 cycles of denaturation at 95°C for 45 s, annealing at 53°C for 45 s, extension at 72°C for 45 s followed by a final extension period at 72°C for 10 min. The PCR mixture for Archaea was the same as that for bacteria with the respective archaeal primers. Protocol amplification for Archaea was: initial denaturation for 2 min at 94 °C; 30 cycles of denaturation at 94°C for 1 min, annealing at 55°C for 1 min, extension at 72°C for 1 min; followed by a final extension at 72°C for 10 min PCR products of each soil sample were amplified in triplicate with a 25 cycle–based protocol for Bacteria, and 30 cycle–based protocol for Archaea. Amplicons were purified using the GFX™ PCR DNA and Gel Band Purification Kit (GE Healthcare, UK). Each library was quantified using in a NanoDrop™ 3300 fluoroespectrometer (Thermo Fisher Scientific Inc., Suwanee, GA, USA) and mixed in equal amounts. Sequencing was done unidirectionally by Macrogen Inc. (Seoul, Korea) using the Roche 454 GS–FLX Titanium (Roche 454 Life Sciences, Branford, CT, USA).

### Pyrosequencing reads processing

Sequences were processed for quality, barcode sorting and denoising was done through the QIIME pyrosequencing pipeline, version 1.9 (http://www.qiime.org/). Briefly, reads shorter than 250 bp, with quality Phred scores (Q score) < 20, or containing errors in adaptors and primers were discarded. One mismatch was allowed in the barcode sequence. Denoising of the reads was done with the script *denoise_wrapper.py* using the barcode–sorted libraries and the standard flowgram format (SFF) files (Reeder and Knight, 2010). Singletons were not included in the sequences to be analyzed. Sequences are available at the Sequence Read Archive (SRA) under the accession number SRP041362, SRP068519, SRR4298851-SRR4298883.

The screened sequences were used to determine *de novo* operational taxonomic units (OTUs) at 97% cut–off with the script *pick_de_novo_otus.py*. One representative sequence for each OTU was chosen, and potentially chimeric sequences were detected using ChimeraSlayer (Haas et al., 2011) and removed from the representative sequences data set.

### Taxon–based and phylogenetic analyses

The taxonomic assignments were done with the naïve Bayesian rRNA classifier from the Ribosomal Data Project (http://rdp.cme.msu.edu/classifier/classifier.jsp) at a confidence threshold of 80% (Wang et al., 2007). The obtained biological observation matrix (BIOM) table was normalized by rarefying to 1, 270 reads per sample, to avoid bias in diversity analysis by differences in sampling-sequencing effort using the single_rarefaction.py script within QIIME pipeline. Diversity (Shannon, Simpson, and phylogenetic diversity indices) and species richness estimators (Chao1) were calculated using the rarified datasets within QIIME pipeline with the script alpha_diversity.py. The relative abundances were calculated for OTU and genus-taxonomic level in each sample.

The representative sequence data set was aligned at a minimum percent sequence identity of 75% using PyNast (Caporaso et al., 2010a). Sequences that could not be aligned were removed. Neighbor joining phylogenetic trees were constructed with evolutionary distances obtained by a Maximum Likelihood approach within the QIIME pipeline (Caporaso et al., 2010b). Phylogenetic information was also used to calculate pairwise UniFrac distance matrices using weighted data within QIIME.

The analysis of variance (ANOVA) to determine the effect of flooding on the relative abundance of the archaeal and bacterial groups (phylum, class, order, family, and genus) was based on the least significant difference using the general linear model procedure PROC GLM (SAS Institute, 1989). The effect of flooding on soil characteristics was also analyzed using PROC GLM (SAS Institute, 1989).

Abundance of the different archaeal and bacterial taxonomic levels was explored separately with a principal component analysis (PCA) using PROC FACTOR (SAS Institute, 1989). A canonical correlation analysis (CCA) was used to study the degree of relationship between the abundance of the different archaeal and bacterial groups, and the soil characteristics. The CCA was done using the PROC CANCORR of the SAS statistical package (SAS Institute, 1989). A Spearman rank coefficient (*r*) for correlation between soil bacterial communities, i.e., indicator operational taxonomic units (OTU) at a similarity threshold of 97% (OTU_97_), and physicochemical soil properties, i.e., pH, electrolytic conductivity (EC), water holding capacity (WHC), and clay and sand content, was calculated in R (http://www.inside-r.org/packages/cran/vegan/docs/bioenv; Clarke and Ainsworth, 1993).

Cluster analyses were done using the UniFrac pair- wise distance matrix using Unweighted Pair Group Method with Arithmetic Mean (UPGMA). Robustness determination of individual UPGMA clusters was performed by comparing rarefied UPGMA trees to either (full or consensus) tree for jackknife support of tree nodes.

## Results

### Soil characteristics

The EC of the soil decreased sharply from 157.8 dS m^−1^ in the unflooded soil to 47.3 dS m^−1^ after flooding the soil once and to 1.7 dS m^−1^ after 10 floodings (*p* < 0.0001) (Supplementary Table S2). Flooding did also affect the particle size distribution. The clay content decreased gradually from 430 to 270 g kg^−1^ soil and the sand content increased gradually from 260 to 500 g kg^−1^ soil (*p* < 0.01). The pH did not change over time while WHC fluctuated with flooding, but did not show a clear pattern.

### Archaeal community structure

Overall, 144,702 high quality archaeal ribosomal sequences were *de novo* clustered in 1, 517 different OTU's. The number of archaeal OTUs obtained for the number of sequences retrieved after each flooding was similar (Supplementary Figure S1A). Increasing the number of sequences retrieved from soil would only marginally increase the number of archaeal OTUs. The number of archaeal species, Shannon, Simpson, and PD indices increased significantly after the first flooding compared to soil flooded more than once (*p* < 0.01) (Supplementary Table S3). The diversity and archaeal species richness (Shannon, Simpson and PD indices, and Observed species and Chao (1) tended to increase with increased floodings, but not significantly.

Three archaeal phyla were detected in the soil, i.e., Crenarchaeota with a mean relative abundance of 0.007%, Thaumarchaeota with 0.50% and Euryarchaeota with 91.8%. The remaining sequences remained unassigned. Phylotypes belonged to six classes, eight orders, 12 families, and only 26 genera. Halobacteriaceae were the dominant family with a relative sequence abundance that ranged from 79.2 to 96.9% with *Natronococcus* the dominant genus with a relative abundance that ranged from 12.1 to 21.8%. More than half of the phylotypes could not be assigned to a genus.

**Figure 2:**
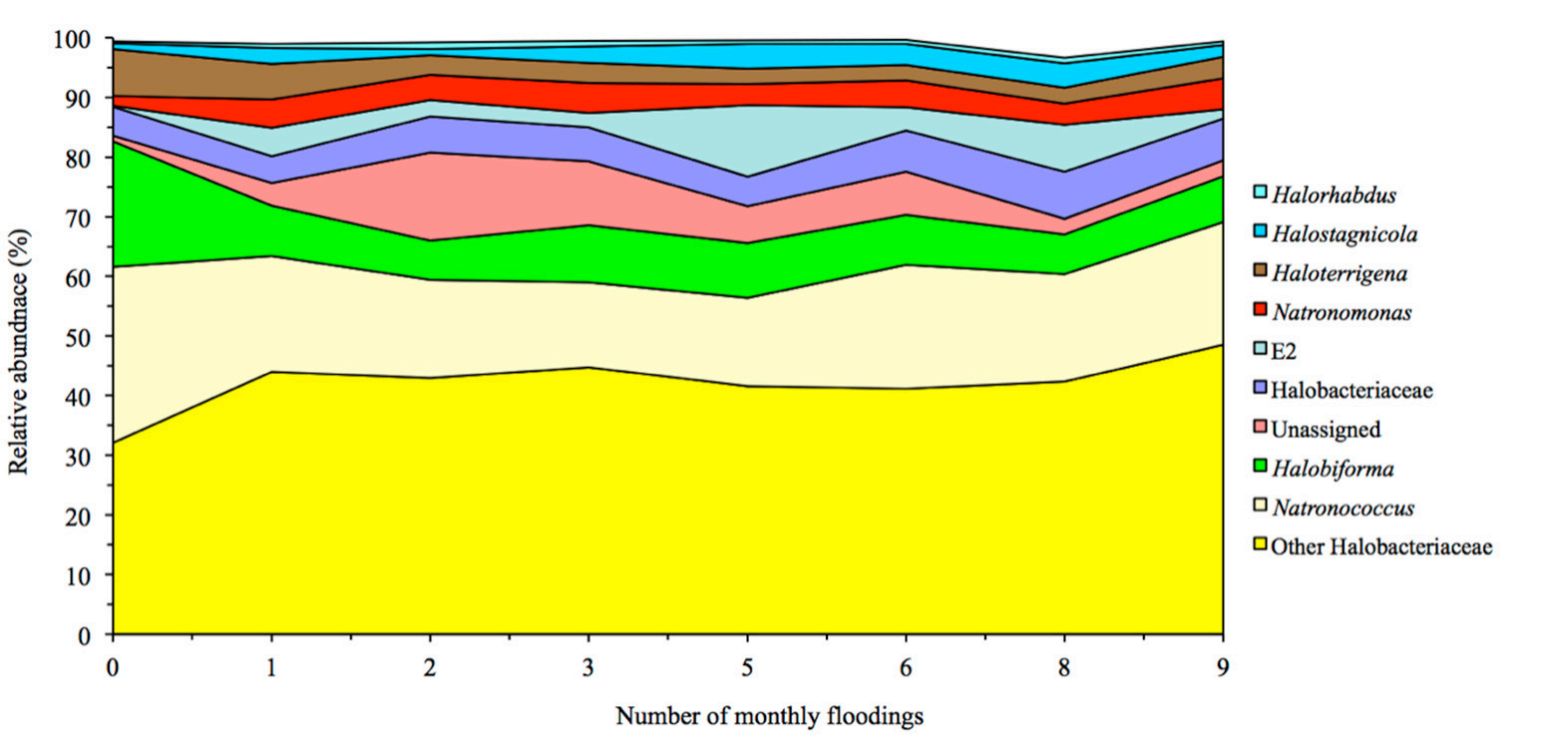
**Changes in the relative abundance of the most abundant archaeal groups in soil columns following 10 months of flooding treatment**.

**Figure 3:**
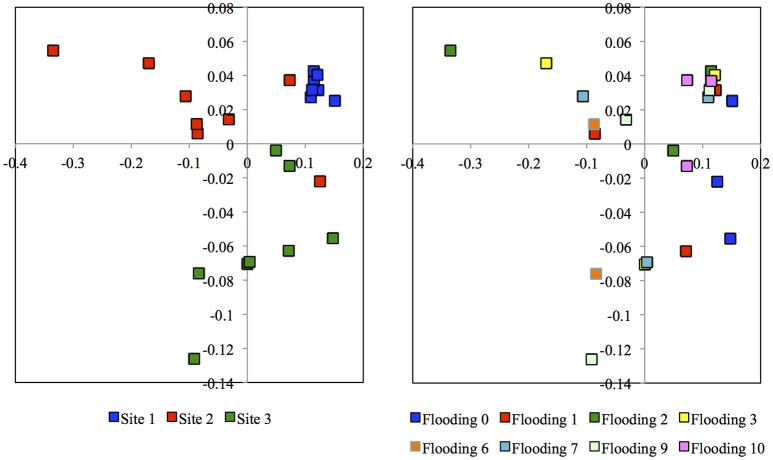
**Principal coordinate analysis of the weighted UniFrac distances of the archaeal OTUs clustered at a 97% similarity**. Archaeal communities were from three sampling sites and never flooded (Flooded 0), flooded once (Flooded 1), flooded twice (Flooded 2), three times (Flooded 3), six times (Flooded 6), seven times (Flooded 7), nine times (Flooded 9), or ten times (Flooded 10). The first principal coordinate (PC1) explained 68% of the variation and PC2 11%.

Flooding the soil had only a limited effect on the archaeal population (Figure 2). Only the relative sequence abundance of phylotypes belonging to *Haloferax* was significantly higher in soil flooded 10 times (0.16%) than in the other soils (< 0.03%). Consequently, the PCoA or PCA did not separate the archaeal community structure independent of the archaeal taxonomic level considered, i.e., phylum, class, order, family, genus, or OTU's (Figure 3, Supplementary Figures S2, S3). The archaeal structure appeared to be more defined by the sampling site as the PCA separated the different sampling sites. Sampling site 3 was generally characterized by a more negative PC1, e.g., a higher relative abundance for *Halobiforma, Halostagnicola, Methanospirillum* and *Natronococcus*, and sampling site 1 by a positive PC1, e.g., a higher relative abundance for *Halorhabdus, Natronomonas*, and other Halobacteriaceae.

The spearman rank coefficient (*r*) between the soil archaeal communities, i.e., indicator operational taxonomic units (OTU) at a similarity threshold of 97% (OTU_97_), and physicochemical soil properties, i.e., pH, electrolytic conductivity (EC), water holding capacity (WHC), and clay and sand content was low. The environmental variable with the best correlation to the archaeal community was EC with a negative r of −0.0008 (Table 1).

**Table 1:** **Spearman rank coefficient (***r***) for correlation between soil microbial communities, i.e., indicator operational taxonomic units (OTU) at a similarity threshold of 97% (OTU_**97**_), and physicochemical soil properties, i.e., pH, electrolytic conductivity (EC), water holding capacity (WHC), and clay and sand content**.

	**BioEnv factor^a^**	***r* coefficient**
Bacterial communities based on OTU_97_	Clay	0.438
Archaeal communities based on OTU_97_	EC	−0.0008

a *Environmental factors included pH, EC, WHC, clay, and sand*.

The CCA, however, did show an effect of flooding on the archaeal structure (Supplementary Figure S4). The unflooded soil was characterized by a negative CC1, i.e., a higher EC and a higher relative sequence abundance of *Halobiforma, Haloterrigena*, Halobacteriaceae, *Methanoculleus*, and *Natronococcus*, and separated clearly from the flooded soils characterized by a positive CC2, i.e., a higher relative abundance of Halorhabdus, *Halostagnicola*, and *Natronomonas*. The soils flooded 9 and 10 times characterized by a negative CC2, i.e., a higher sand content and a higher relative abundance of pGrfC26, Candidatus Nitrososphaera, *Haloferax*, MSP41, other Methanomicrobiaceae, were also separated from the soils flooded 1, 2, 3, 6, and 7 times characterized mostly by a positive CC2, i.e., a higher pH and WHC, and a higher relative abundance of unassigned sequences, *Halorubrum, Methanolobus*, MHVG, *Halosimplex*, Cenarchaeaceae, *Methanosaeta, Nitosopumilus*, XKL75. The UPGMA did not show any clear separation pattern, however archaeal OTUs grouped according to sampling site, rather than flooding (Supplementary Figure S5).

### Bacterial community structure

Overall, 254,000 sequences of 6, 403 different bacterial OTU's were retrieved from the soil. The number of bacterial OTUs obtained from the number of sequences retrieved after each flooding was similar so that a comparison was possible (Supplementary Figure S1B). Increasing the number of sequences retrieved from soil would only marginally increase the number of bacterial OTUs. The number of bacterial species, and the Chao1, Shannon, Simpson, and PD indices did not change after the first flooding or thereafter (*p* < 0.01) (Supplementary Table S3). No clear pattern emerged in the number of bacterial species, and alpha indices with increased floodings.

**Figure 4:**
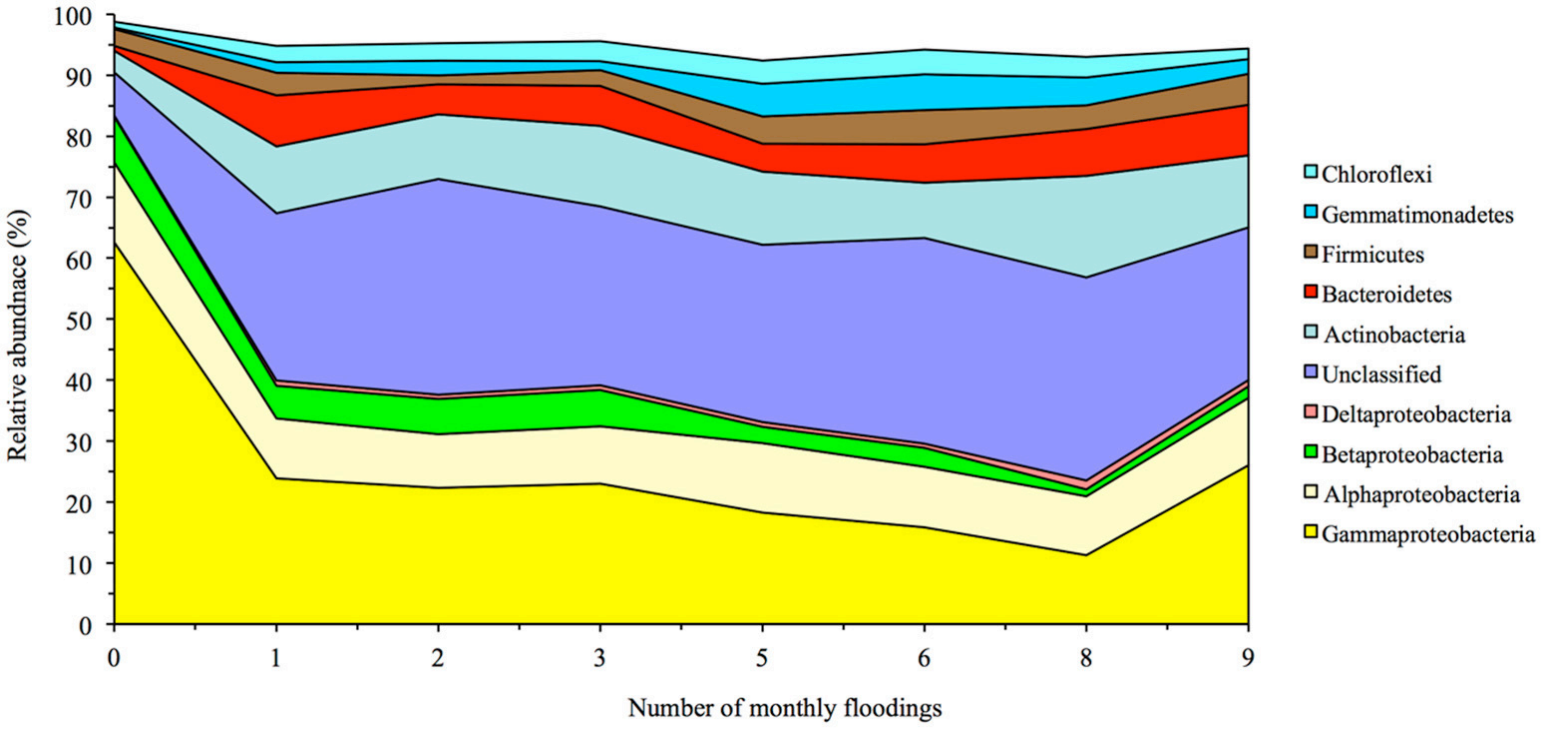
**Effect of flooding on the relative abundance of the most abundant bacterial phyla and classes of the Proteobacteria**.

Twenty-nine bacterial phyla were detected in the soil with the Proteobacteria the most abundant (relative abundance of 83.4%; Figure 4, Supplementary Table S4). Flooding the soil had a strong effect on the bacterial community structure. Flooding the soil once decreased the relative sequence abundance of the Acidobacteria, Chlorobi and Proteobacteria. The relative abundance of the Proteobacteria showed the largest drop and decreased significantly from 83.4% in the unflooded soil to 40.0% after the first flooding (*p* < 0.0001). This decrease was mostly due to a drop in the relative abundance of the Gammaproteobacteria as the relative abundance of Halomonadaceae (Oceanospirillales) dropped from 23.9% in the unflooded soil to 3.0% in the soil flooded once, Pseudomonadaceae from 5.4 to 0.9% and Xanthomonadaceae from 4.8 to 0.5%. The relative sequence abundance of the Rhizobiales (Alphaproteobacteria) dropped also from 11.4% in the flooded soil to 2.7% in the flooded soil, but that of the Rhodobacterales (also Alphaproteobacteria) increased sharply from 0.5 to 6.3%.

The relative abundance of most other bacterial phyla, i.e., Actinobacteria, Bacteroidetes, Gemmatimonadetes, Planctomycetes, TM7, Verrucomicrobia, and [Thermi], and the unassigned phylotypes showed an opposite pattern and increased after the first flooding. For instance, the relative abundance of the Actinobacteria (mostly Nitriliruptorales) increased significantly from 3.6% in the unflooded soil to 11.0% in the soil flooded once and that of the Bacteroidetes [mostly (Rhodothermales) and Cytophagales] from 0.7 to 8.4%.

**Figure 5:**
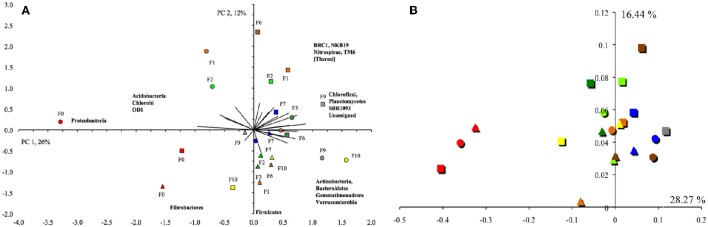
**(A)** Principal component analysis with the different bacterial phyla. The first principal component (PC1) explained 26% of the variation and PC2 12%. Principal component analysis with the different archaeal groups from the first sampling site at Texcoco soil never flooded (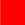), flooded once (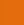), flooded twice (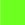), three times (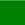), six times (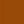), seven times (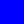), nine times (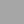) or ten times (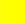), second sampling site never flooded (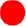), flooded once (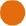), flooded twice (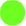), flooded three times (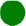), six times (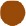), seven times (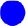), nine times (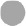) or ten times (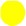), and third sampling site never flooded (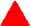), flooded once (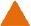), flooded twice (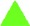), flooded three times (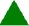), six times (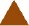), seven times (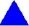), nine times (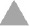) or ten times (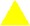). The first principal component (PC1) explained 22% of the variation and PC2 17%. **(B)** Principal coordinate analysis of the weighted UniFrac distances of the bacterial OTUs clustered at a 97% similarity. Legends of the figure are the same as **(A)**.

Consequently, the PCA and PCoA separated the unflooded soil clearly from the flooded soils (Figure 5). The unflooded soil was characterized by a negative PC1, e.g., higher relative abundance of Proteobacteria, and a small positive or negative PC2, e.g., a higher relative abundance of Fibrobacteres and Firmicutes. The PCA did not separate the flooded soils from each other. Contrarily, the CCA did (Supplementary Figure S6). The unflooded soil was found in the lower right quadrant, the soil flooded once or twice in the lower left quadrant and the soil flooded ≥6 times in the upper left or right quadrant. The unflooded soil with a higher EC, and silt and clay content had a larger relative abundance for the Chlorobi, Cyanobacteria and Proteobacteria than the soils flooded one or twice characterized by a larger relative abundance for the BRC1 and Fibrobacteres. The soils flooded at least six times with a higher sand content were characterized by a larger relative abundance for most other bacterial phyla, such as the Actinobacteria, Bacteroidetes, Chloroflexi, Firmicutes, Gemmatimonadetes, and Verrucomicrobia. The UPGMA did not show any clear separation pattern of bacterial OTUs (Supplementary Figure S7).

The Spearman rank coefficient (*r*) between the soil bacterial communities, i.e., indicator operational taxonomic units (OTU) at a similarity threshold of 97% (OTU_97_), and physicochemical soil properties, i.e., pH, electrolytic conductivity (EC), water holding capacity (WHC), and clay and sand content was higher than for Archaea. The environmental variable with the best correlation to the bacterial community was clay content with a positive r of 0.438 (Table 1).

## Discussion

### Soil characteristics

In a previous experiment the EC dropped from 59.2 to 21.3 dS m^−1^ when flooding soil of the former lake Texcoco twice (Dendooven et al., 2015). Flooding the soil in the field with effluent and installing a drainage system also succeeded in decreasing EC from 39.9 dS m^−1^ in the undrained soil to 12.6 dS m^−1^ after 8 years (Luna-Guido et al., 2000).

In previous experiments, the particle size distribution of soil from the former lake bed was highly variable. For instance, the clay content was 440 g kg^−1^ in the undrained soil and decreased to 240 g kg^−1^ in soil flooded and drained for 8 years, while the sand content increased from 400 to 720 g kg^−1^ (Luna-Guido et al., 2000). We speculated that this was due to the intrinsic variability of the former lake bed or flooding the soil and draining it washed the clay particles out while the sand particles remained. The experiment reported here under controlled conditions confirmed that flooding the soil washed out clay particles as the clay content decreased from 430 to 270 g kg^−1^, while the sand content increased from 260 to 500 g kg^−1^.

### Archaeal community

Euryarchaeota (91.82 ± 0.09%) dominated among Archaeal phylotypes in soil of the former lake Texcoco. They often dominate in soil (Schneider et al., 2015), but also in some Oceanic basins, e.g., north-western Black Sea (Stoica and Herndl, 2007). Euryarchaeota have been found also in greater relative abundance in previous experiments. More than 95% of the clones analyzed by Valenzuela-Encinas et al. (2008) in a soil with EC 159 dS m^−1^ and pH 10.5 were affiliated with members of the family Halobacteriaceae belonging to the phylum Euryarchaeota. Navarro-Noya et al. (2015) also found Euryarchaeota to dominate the soil with EC 157.2 dS m^−1^ and pH 10.2.

Phylotypes belonging to three different classes of the Euryarchaeota were detected in the Texcoco soil: Halobacteria (87.61 ± 0.11%), which survive extreme salinity, Methanomicrobia (0.06 ± 0.001%), which produce methane and contain halophiles, and Thermoplasmata (4.16 ± 0.04%), extremely thermophilic aerobes and anaerobes. Most of the archaeal genera of the Halobacteria detected in the Texcoco soil have been described in high-pH soda lakes, e.g., *Natronococcus, Natronomonas, Halorubrum*, and *Halobiforma* (Jones et al., 1998; Grant and Sorokin, 2011) and in waters rich in Mg^2+^, e.g., *Natronococcus, Halorhabdus*, and *Natronomonas* (Oren, 2002; Rhodes et al., 2012). *Natronococcus* (18.28 ± 0.08%) was the most abundant genus in the Texcoco soil together with *Halobiforma* (9.08 ± 0.08%). Contrarily, Najjari et al. (2015), while studying halophilic Archaea (Class Halobacteria) in Tunisian endorheic salt lakes and sebkhet systems (*n* = 23), reported a maximum relative abundance of 1.6% for *Natronococcus*. The genus was absent often from samples so they considered it a rare genus with occasional low abundance. In the Texcoco soil *Natronococcus* was favored by the high pH rather than salinity. Najjari et al. (2015) considered *Halobiforma* a moderately abundant genera reaching a relative abundance of 18.8%, similar as found in the unflooded soil, in a sediment sample with 6.7% salinity. They considered *Halogranum* and *Halorubrum* as consistently abundant genera, but in this study the first was detected in only one soil sample (relative sequence abundance of 0.026%) and the relative abundance of the latter remained low (≤ 0.052%). It is possible that *Halobiforma* was better adapted to a higher salt content (21.0% in the unflooded soil) as its relative abundance more halved after one flooding (8.5%) and remained low afterwards. Some phylotypes belonging to this genus require salt for growth. For instance, *Halobiforma lacisalsi*, isolated from a salt lake in China requires >1.7 M NaCl and grows optimally 2.6 and 4.3 M NaCl (Xu et al., 2005). *Natronococcus* (29.53% in the unflooded soil) and *Haloterrigena* (7.9% in the unflooded soil) were also better adapted to higher salt content, but the effect of decreasing salt content on the latter was slower and the first seemed to adapt to a decreasing salt content as its relative abundance increased again after ten floodings. It must be noted that in this study, like in the majority of similar studies, we did not measure absolute abundance, but relative abundance of phylotypes (Dillon et al., 2013).

The relative abundance of the Thaumarchaeota was low in the Texcoco soil. This phylum is associated with ammonia-oxidizing organisms that gain energy by chemolithotrophic catabolism and are autotrophic or obligate mixotrophic metabolism (Könneke et al., 2005; Qin et al., 2014; Sintes et al., 2016). They are found in soils, marine environments, on the surface of weathering rocks and thermal waters and are abundant in pneumatophore-associated soil regardless of water content in a tropical mangrove ecosystem (Stieglmeier et al., 2014; Dong et al., 2015; Loganathachetti et al., 2016) and *Thaumarchaeota* dominate the mesopelagic and bathypelagic zone in deep ocean waters (Zhu et al., 2016). Phylotypes belonging to *Nitrosopumilus* and Candidatus Nitrosphaera (Thaumarchaeota) were detected in the Texcoco soil. *Nitrosopumilus maritimus* is autotrophic and a nitrifier isolated first from a marine environment (Walker et al., 2010). Little information exists about Candidatus Nitrosphaera, but it was one of the most abundant Archaea in two soils from the Amazonian region (Taketani and Tsai, 2010).

*Crenarchaeota* were the least abundant phylogroup in the Texcoco soil. *Crenarchaeota* are abundant in the oceans, although some have been found in soil (Ramos-Vera et al., 2011) as in this study. They were thought to be sulfur-dependent extremophiles, but recent studies indicating that these organisms may be the most abundant Archaea in some marine environments, e.g., sediments, deep ocean, or subsurface (Karner et al., 2001; Kirchman, 2012).

Although flooding the soil decreased the EC sharply, fewer archaeal groups were affected by changes in salt content than bacterial groups. There are different possible explanations for this observation. First, the change in EC was too short to alter the archaeal community structure. This would be in contrast to the bacterial community structure that was altered within the first flooding. The archaeal community was more resistant to changes in EC than the bacterial community and only after an extended period of time will the archaeal community structure change. Second, EC was not the driving force and the archaeal community was controlled by pH or organic matter, that did not change with flooding. Tripathi et al. (2015) found that pH correlated with the composition of the archaeal community in temperate and tropical soils, but also the biome, while Canfora et al. (2015) reported that organic matter in soil favored their diversity. Navarro-Noya et al. (2015) reported that differences in pH better explained the composition of the archaeal community in haloalkaline Texcoco soils. Third, it has to be remembered that the number of archaeal taxonomic groups was lower than the number of bacterial taxonomic groups. This could also explain why less archaeal groups were affected by decreases in salt content than bacterial groups.

### Bacterial community

Bacterial genera belonging to the Alphaproteobacteria, Firmicutes, Gammaproteobacteria, Bacteroidetes, and Cyanobacteria are found in soda brines with salinities up to 250 g L^−1^ (Vavourakis et al., 2016). In the unflooded Texcoco soil, these bacterial groups also dominated, although the relative abundance of the Actinobacteria and Betaproteobacteria was also high. Oceanospirillales (*Halomonas*), Pseudomonadales, Phyllobacteriaceae (Rhizobiales) were the most abundant bacterial orders. In previous studies, Valenzuela-Encinas et al. (2009) found that most of the clones of Gammaproteobacteria were related to microorganisms previously found in soda lakes and in soils. Microorganisms belonging to the Chromatiales found in marine, hypersaline and haloalkaline environments were found in a soil with 159 dS m^−1^ and pH 10.2.

Phylotypes belonging to *Halomonas* have been found to dominate bacterial populations often in extreme saline environments. Vavourakis et al. (2016) reported that the relative abundance of *Halomonas* in a soda lake with salinity 170 g L^−1^ and pH 9.9 was 2%, but 71% in a soda lake with salinity 400 g L^−1^ and pH 10.2. *Halomonas* was not as dominant in this study as its relative abundance was 23.9%. They also reported that the dominant fraction in the soda lake with salinity 170 g L^−1^ and pH 9.9 (~59%) was affiliated with no known order of the Gammaproteobacteria. In this study, 25.2% of the Pseudomonadales was not affiliated with a known family. Unknown phylotypes of *Phyllobacterium* (Phyllobacteriaceae) dominated in the Red Sea brine-seawater interface (54%) (Abdallah et al., 2014), and the Phyllobacteriaceae represented 9.1% in this study. Phylotypes belonging to *Phyllobacterium* species participate in aromatic hydrocarbon mineralization and have been retrieved from marine ecosystems (von der Weid et al., 2008; Shao et al., 2010).

In contrast to the archaeal community structure, flooding the soil once altered the bacterial community structure profoundly, i.e., changes in the relative abundance of some bacterial groups were much larger compared to those of archaeal groups. It has to be remembered that no unflooded soil was kept during the entire experiment and some changes in the relative abundance of the different bacterial might be due to storage and not due to flooding. The decrease in relative abundance was most accentuated for bacterial groups belonging to the Alpha- and Gammaproteobacteria. The relative abundance of the dominant bacterial groups in the soil flooded once more than halved, i.e., Oceanospirillales (*Halomonas*), Pseudomonadales (*Pseudomonas*), Rhizobiales (Phyllobacteriaceae), and Xanthomonadales (other Xanthomonadaceae). They were replaced with phylotypes belonging to different bacterial phyla, e.g., the Cytophagales and [Rhodothermales] (Bacteroidetes), Rhodobacteraceae (Rhodobacterales, Alphaproteobacteria), Nitriliruptoraceae (Nitriliruptorales, Actinobacteria), Deinococcales ([Thermi]), and Gemm-5 (Gemmatimonadetes) as the most important. The relative abundance of each of them increased more than 5-times. It is interesting to notice that some important bacterial groups, e.g., known copiotrophs in soil such as the Actinomycetales and Bacillales, were not affected by flooding the soil.

Ongoing monthly flooding further affected bacterial groups, but the overall effect was smaller. The relative abundance of some groups tended to decrease further, i.e., Oceanospirillales and Pseudomonadales, while that of others, i.e., Rhizobiales and Xanthomonadales, tended to increase again. The relative abundance of the bacterial groups that increased sharply after the first flooding remained similar, e.g., Cytophagales, Deinococcales, Nitriliruptorales, Rhodobacterales, and [Rhodothermales], while that of others increased further, e.g., Gemm-5. Although the relative abundance of the Burkholderiales did not show a sharp drop, after 10 floodings it had dropped 4-fold, i.e., mostly phylotypes belonging to the *Delftia* (Comamonadaceae).

## Conclusions

Flooding the soil reduced the EC from 157.8 to 1.7 dS m−1 and the clay content from 430 to 270 g kg−1, but the pH remained the same. After one flooding event and 1 month in the columns, the bacterial community structure was altered, as the relative abundance of Halomonas, Pseudomonas, Rhizobiales, and Xanthomonadales decreased, while that of the Cytophagales, [Rhodothermales], Rhodobacteraceae, Nitriliruptoraceae, Deinococcales, and Gemm-5 increased. Subsequent flooding of the soil altered the bacterial community structure further, but to a lesser extent than after the first flooding. Changes in the archaeal community structure due to flooding were smaller than changes in the bacterial community structure. The question remained if Archaea were more resilient to changes in soil characteristics or they were metabolic more versatile than Bacteria.

## Author contributions

Ad: Writing of manuscript and Data analysis. LaD: Did the experiment. CD: Participated in the Experiment. YN: Experimental design, Data analysis, and Writing of manuscript. ML: Experimental design and Writing of manuscript. LuD: Conceived the experiment, Experimental design, Writing of manuscript, and Data analysis.

## Funding

Ad, LaD, and CD received grant-aided support from “Consejo Nacional de Ciencia y Tecnología” (CONACyT, Mexico). We thank ABACUS-CONACyT for the use of their computer to denoise the sequences. The research was funded by “Instituto de Ciencia y Tecnología del Distrito Federal” (ICTyDF, Mexico). This research was funded by Centro de Investigación y de Estudios Avanzados del Instituto Politécnico Nacional (Cinvestav), “Apoyo Especial para Fortalecimiento de Doctorado PNPC 2013” and project “Infraestructura 205945” from “Consejo Nacional de Ciencia y Tecnología” (CONACyT, Mexico).

### Conflict of interest statement

The authors declare that the research was conducted in the absence of any commercial or financial relationships that could be construed as a potential conflict of interest.
