# Supplementary figures and images for: Reducing Salinity by Flooding an Extremely Alkaline and Saline Soil Changes the Bacterial Community but Its Effect on the Archaeal Community Is Limited
Source: Front Microbiol. 2017 Mar 27;8:466. doi: 10.3389/fmicb.2017.00466 (PMC5366314; doi:10.3389/fmicb.2017.00466)

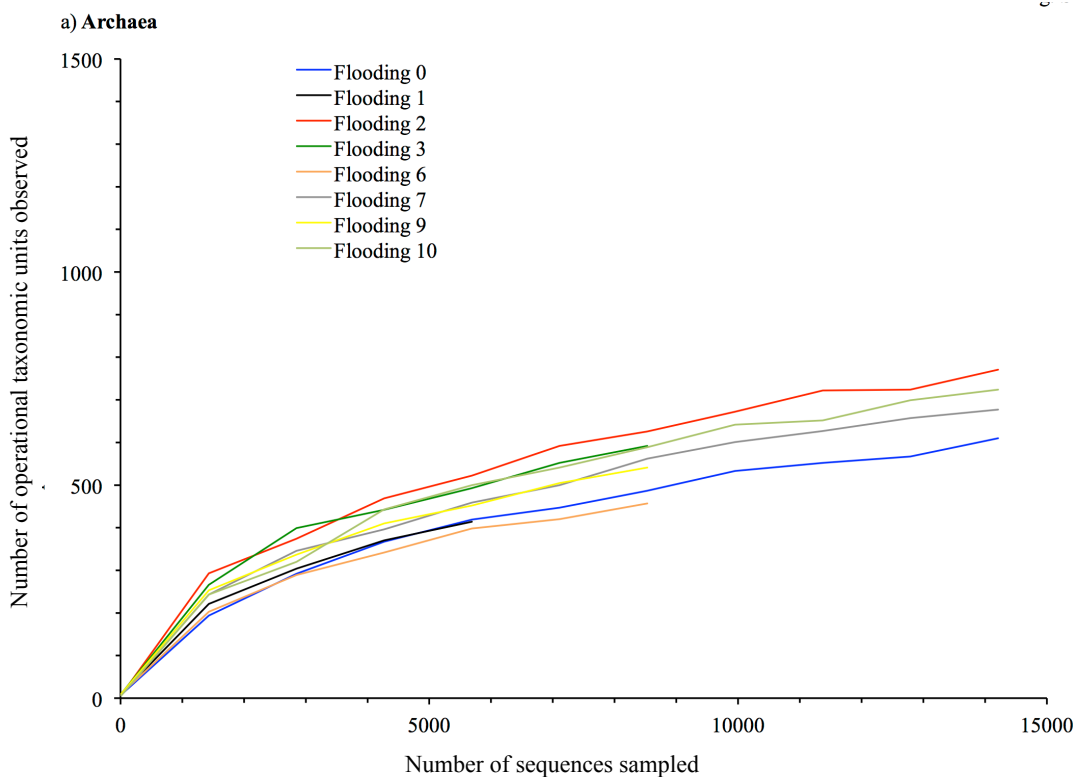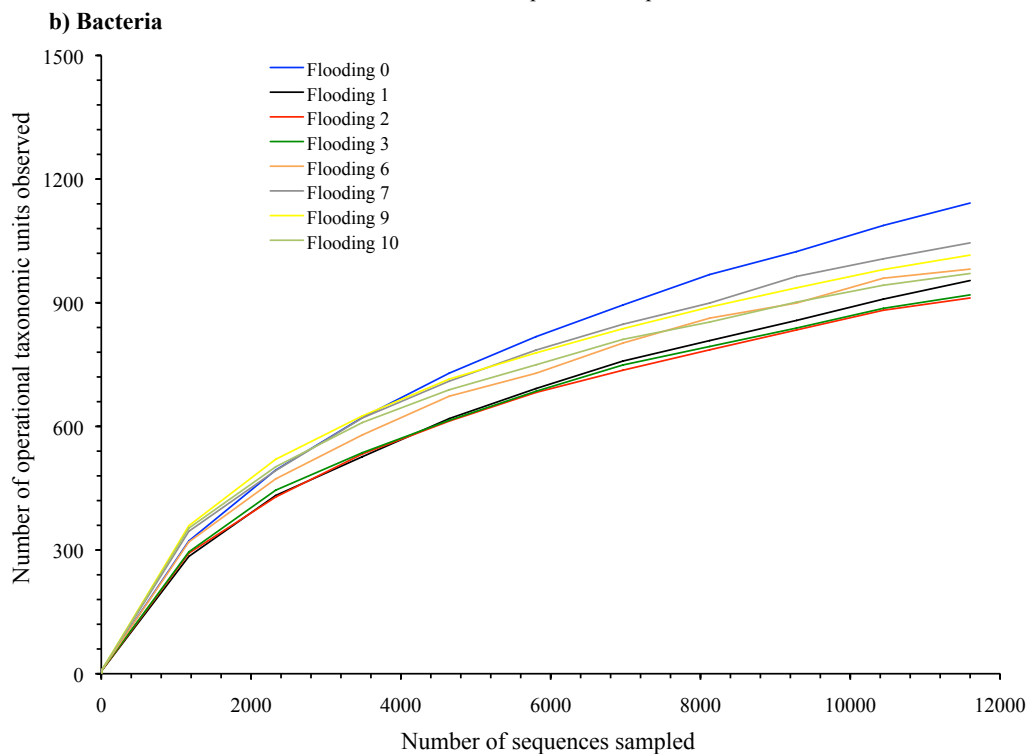

Supplementary Figure S1. Rarefaction curves A) Archaea and B) Bacteria.

Supplement: Supplementary file 1 [file Image1.PDF]
