# Supplementary material for: Reducing Salinity by Flooding an Extremely Alkaline and Saline Soil Changes the Bacterial Community but Its Effect on the Archaeal Community Is Limited
Source: Front Microbiol. 2017 Mar 27;8:466. doi: 10.3389/fmicb.2017.00466 (PMC5366314; doi:10.3389/fmicb.2017.00466)
Supplement: Supplementary file 3 [file Image3.PDF]

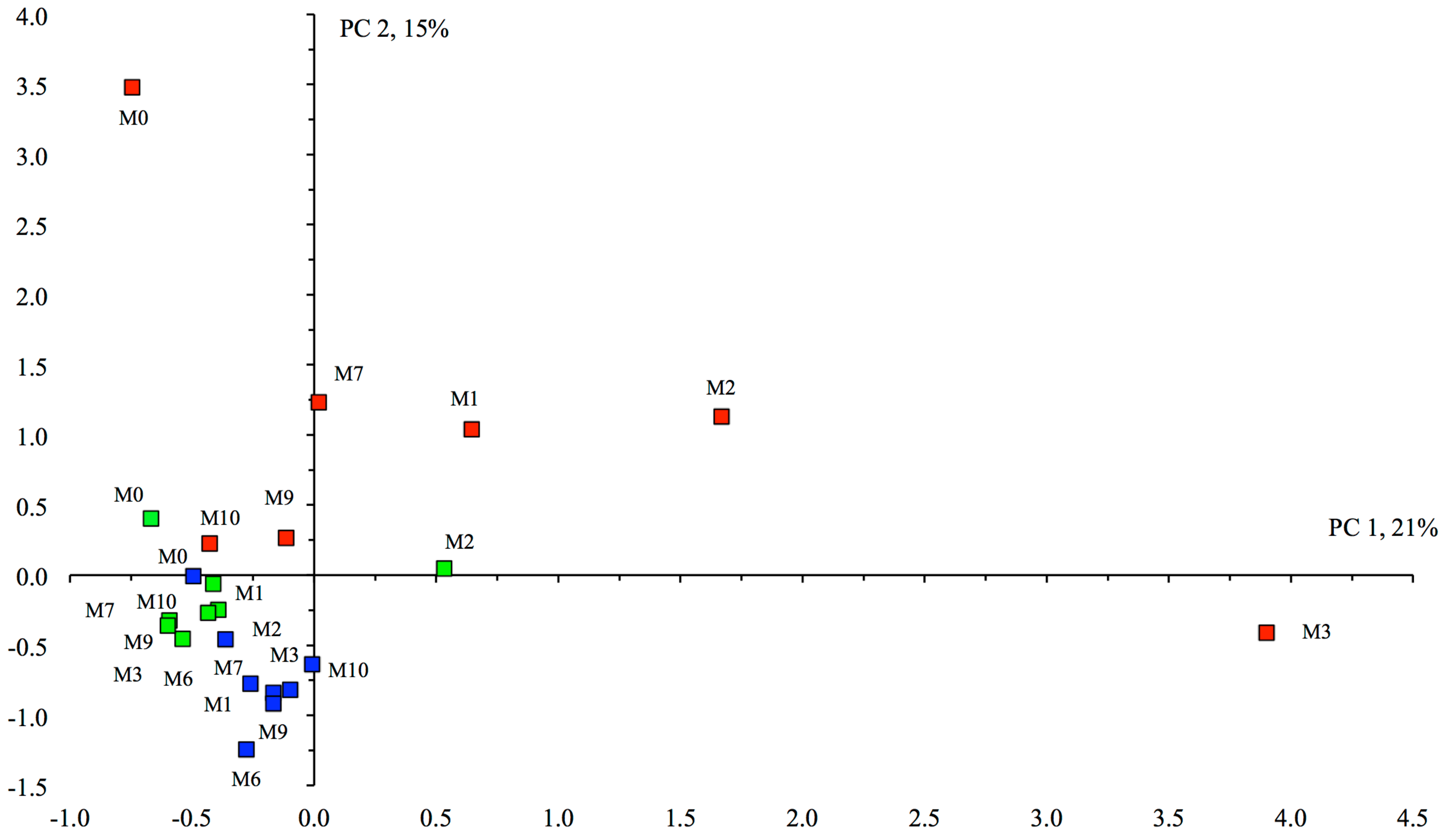

Supplementary Figure S3. Principal component analysis with the different archaeal OTUs from the first sampling site at Texcoco soil never flooded (■), flooded once (■), flooded twice (■), flooded three times (■), six times (■), seven times (■), nine times (■) or ten times (■), second sampling site never flooded (●), flooded once (●), flooded twice (●), flooded three times (●), six times (●), seven times (●), nine times (●) or ten times (●), and third sampling site never flooded (▲), flooded once (▲), flooded twice (▲), flooded three times (▲), six times (▲), seven times (▲), nine times (▲) or nine times (▲). The first principal component (PC1) explained 21% of the variation and PC2 15%.
