# Supplementary material for: Reducing Salinity by Flooding an Extremely Alkaline and Saline Soil Changes the Bacterial Community but Its Effect on the Archaeal Community Is Limited
Source: Front Microbiol. 2017 Mar 27;8:466. doi: 10.3389/fmicb.2017.00466 (PMC5366314; doi:10.3389/fmicb.2017.00466)
Supplement: Supplementary file 4 [file Image4.PDF]

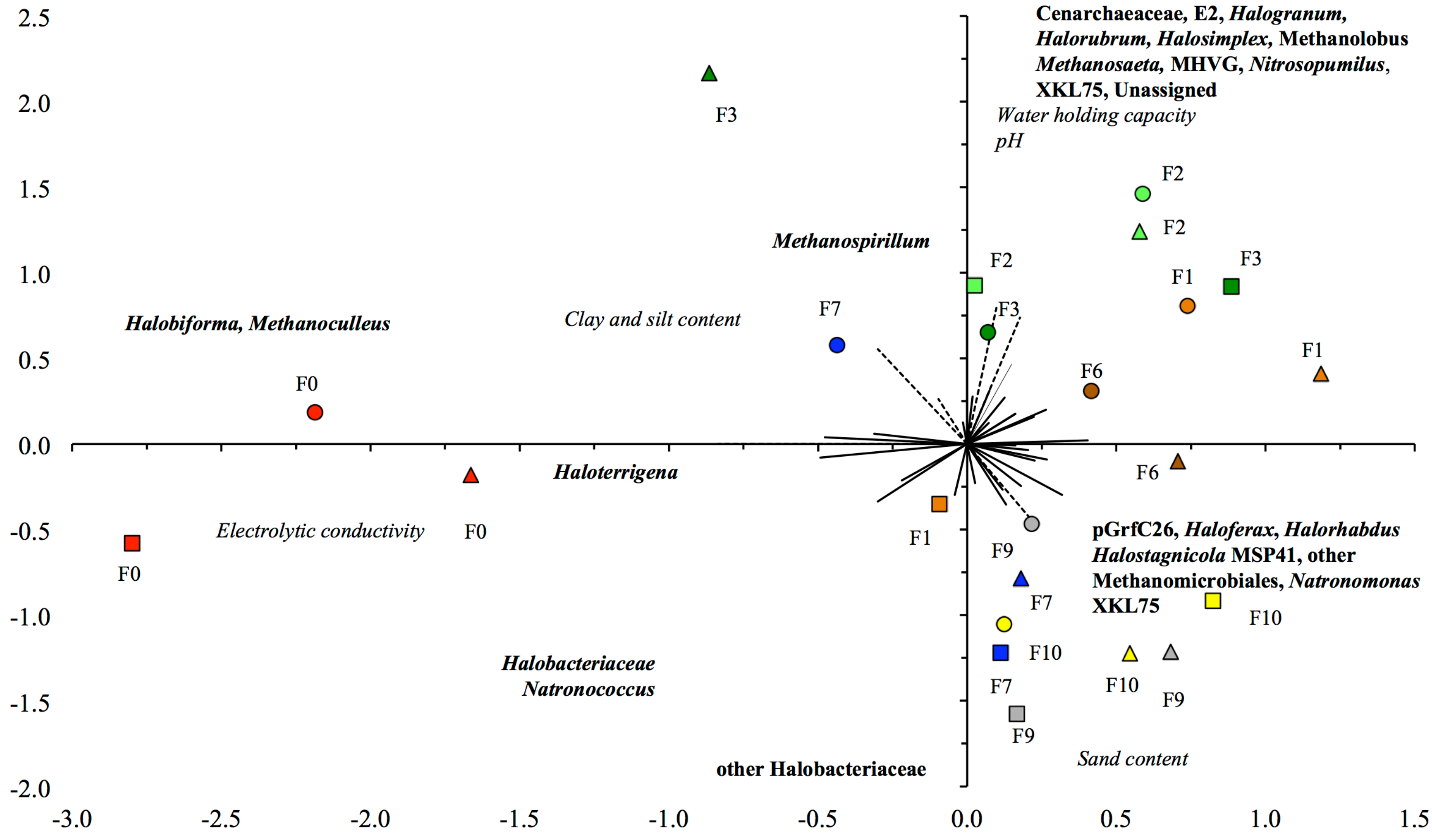

Supplementary Figure S4. Canonical correlation analysis with the different soil characteristics and the archaeal groups from the first sampling site at Texcoco soil never flooded (red square), flooded once (orange square), flooded twice (light green square), three times (dark green square), six times (brown square), seven times (blue square), nine times (grey square) or ten times (yellow square), second sampling site never flooded (red circle), flooded once (orange circle), flooded twice (light green circle), flooded three times (dark green circle), six times (brown circle), seven times (blue circle), nine times (grey circle) or ten times (yellow circle), and third sampling site never flooded (red triangle), flooded once (orange triangle), flooded twice (light green triangle), flooded three times (dark green triangle), six times (brown triangle), seven times (blue triangle), nine times (grey triangle) or ten times (yellow triangle).
