# Supplementary material for: Reducing Salinity by Flooding an Extremely Alkaline and Saline Soil Changes the Bacterial Community but Its Effect on the Archaeal Community Is Limited
Source: Front Microbiol. 2017 Mar 27;8:466. doi: 10.3389/fmicb.2017.00466 (PMC5366314; doi:10.3389/fmicb.2017.00466)
Supplement: Supplementary file 5 [file Image5.PDF]

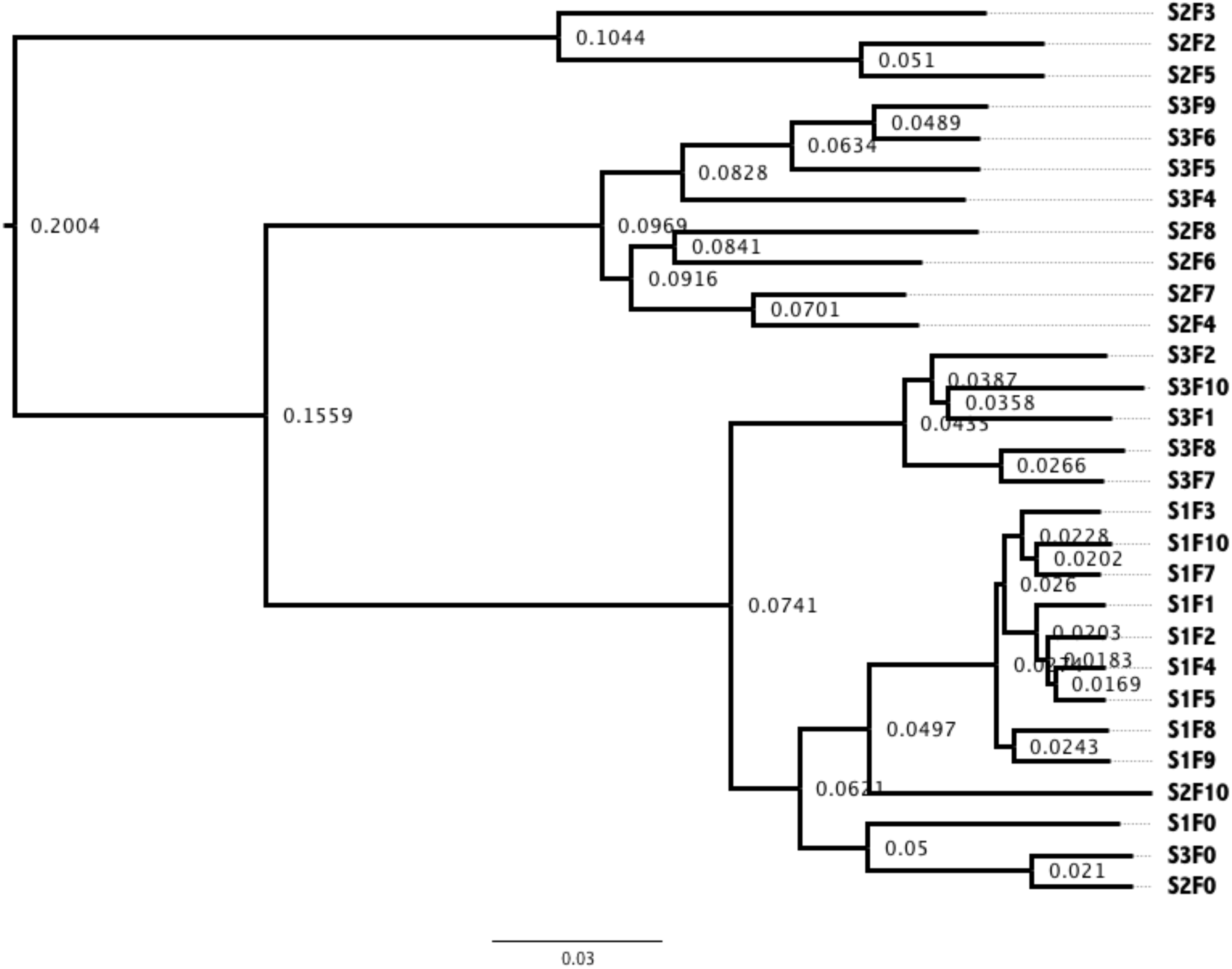

Supplementary Figure S5. UPGMA jackknifed clustering of the UniFrac weighted distances for rarefied 1270-sequence reads of the archaeal communities from alkaline-saline soils of the former lake Texcoco. The scale bar represents 0.03% of divergence UniFrac over all sites analyzed. S1 is site 1, S2 is site 2, S3is site 3. F0-F10 stands for the times the soil was flooded,
