# Supplementary material for: Reducing Salinity by Flooding an Extremely Alkaline and Saline Soil Changes the Bacterial Community but Its Effect on the Archaeal Community Is Limited
Source: Front Microbiol. 2017 Mar 27;8:466. doi: 10.3389/fmicb.2017.00466 (PMC5366314; doi:10.3389/fmicb.2017.00466)
Supplement: Supplementary file 6 [file Image6.PDF]

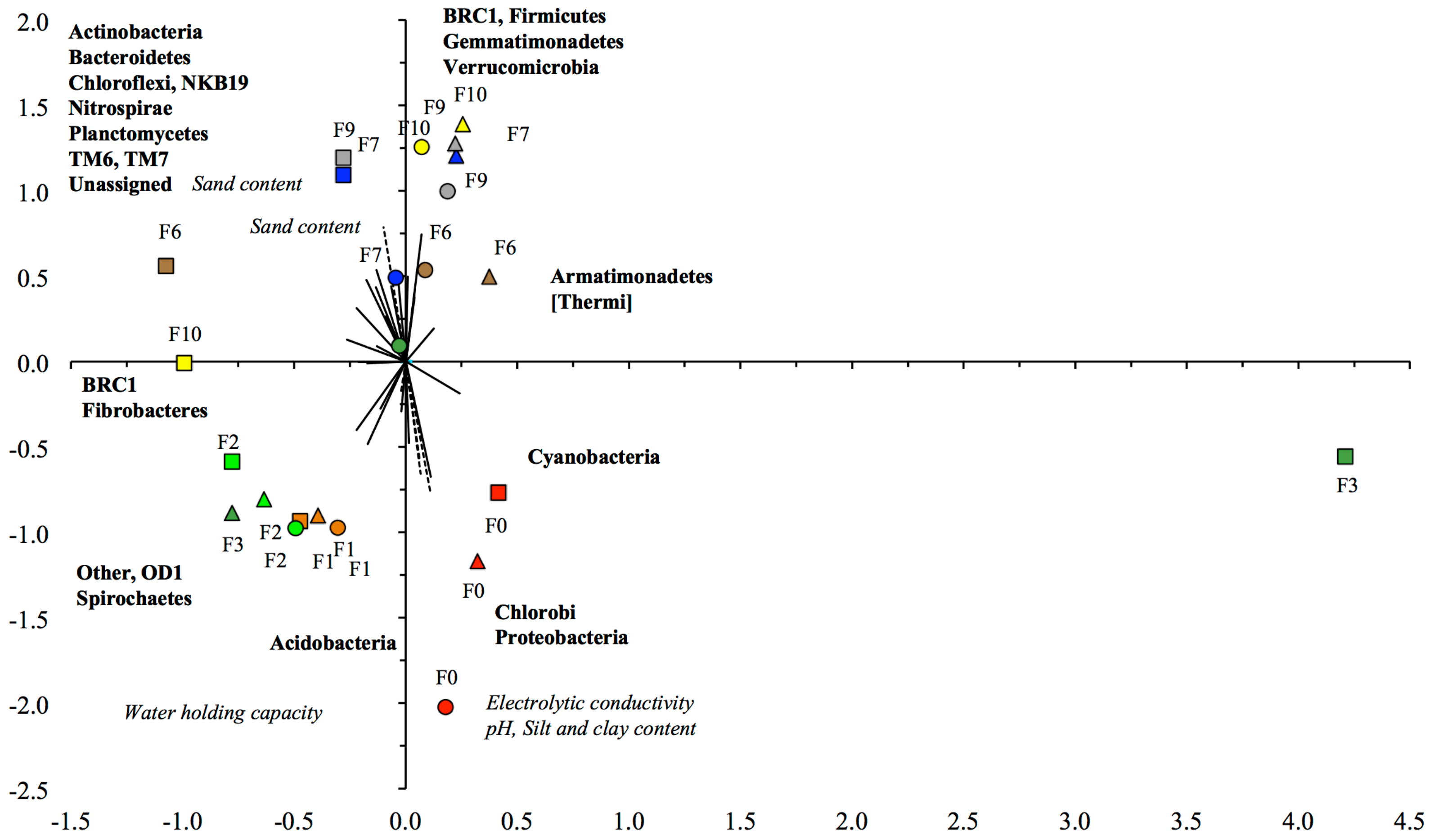

Supplementary Figure S6. Canonical correlation analysis with the different bacterial groups from the first sampling site at Texcoco soil never flooded (■), flooded once (■), flooded twice (■), three times (■), six times (■), seven times (■), nine times (■) or ten times (■), second sampling site never flooded (●), flooded once (●), flooded twice (●), flooded three times (●), six times (●), seven times (●), nine times (●) or ten times (●), and third sampling site never flooded (▲), flooded once (▲), flooded twice (▲), flooded three times (▲), six times (▲), seven times (▲), nine times (▲) or ten times (▲).
