# Supplementary material for: Reducing Salinity by Flooding an Extremely Alkaline and Saline Soil Changes the Bacterial Community but Its Effect on the Archaeal Community Is Limited
Source: Front Microbiol. 2017 Mar 27;8:466. doi: 10.3389/fmicb.2017.00466 (PMC5366314; doi:10.3389/fmicb.2017.00466)
Supplement: Supplementary file 8 [file Table1.PDF]

Supplementary Table 1. Location and initial characteristics of the former lake Texcoco.

Site	Location	EC [a](dSm-1)	pH	Clay	Silt	Sand	Textural Classification
S1	19º 30.800’’ **N** 98º 59.419’’ **W**	157 [b]	10.2	478	131	391	Clay
S2	19º 30.785’’ **N** 98º 59.419’’ **W**	143	10.0	332	92	576	Sandy Clay loam
S3	19º 30.797’’ **N** 98º 59.438’’ **W**	139	10.3	332	216	462	Sandy Clay loam

[a] Electrolytic conductivity
[b] Mean of triplicates (*n*=3).
