# Supplementary material for: Reducing Salinity by Flooding an Extremely Alkaline and Saline Soil Changes the Bacterial Community but Its Effect on the Archaeal Community Is Limited
Source: Front Microbiol. 2017 Mar 27;8:466. doi: 10.3389/fmicb.2017.00466 (PMC5366314; doi:10.3389/fmicb.2017.00466)
Supplement: Supplementary file 9 [file Table2.PDF]

Supplementary Table S2. Characteristics of soil of the former Lake Texcoco that was never flooded and soil flooded monthly, drained freely and incubated at approximately 50% of water holding capacity for ten months.

Times flooded	EC [a] (dS m^{-1})	pH	WHC [b]	Particle size distribution Clay	Silt	Sand
			(g kg^{-1} soil)			
0	157.8 [c] A [d]	10.3 A	770 B	430 A	310 A	260 B
1	47.3 B	10.3 A	880 AB	400 AB	350 A	250 B
2	14.1 C	10.4 A	1150 AB	380 AB	250 A	370 AB
3	8.3 C	10.4 A	1280 A	360 AB	250 A	390 AB
6	5.0 C	10.4 A	850 AB	330 AB	190 A	480 AB
7	3.3 C	10.3 A	1010 AB	320 AB	170 A	510 A
9	2.3 C	10.2 A	870 AB	300 AB	200 A	490 AB
10	1.7 C	10.2 A	840 AB	270 B	220 A	500 A
MSD [e]	25.2	0.2	450	130	200	240
F value	109.60	4.56	3.68	4.81	1.73	4.78
P value	<0.0001	0.0146	0.0146	0.0044	0.1725	0.0046

[a] EC: Electrolytic conductivity, [b] WHC: Water holding capacity, [c] Mean of three plots (*n* = 3), [d] Values with the same capital letter are similar in the flooded soils, i.e. within the column, [e] MSD: Minimum significant difference at 5 % (SAS Institute, 1989).
