# Supplementary material for: Reducing Salinity by Flooding an Extremely Alkaline and Saline Soil Changes the Bacterial Community but Its Effect on the Archaeal Community Is Limited
Source: Front Microbiol. 2017 Mar 27;8:466. doi: 10.3389/fmicb.2017.00466 (PMC5366314; doi:10.3389/fmicb.2017.00466)
Supplement: Supplementary file 10 [file Table3.PDF]

Supplementary Table S3. Alpha diversity of the Archaea and Bacteria in soil of the former Lake Texcoco that was never flooded and soil flooded monthly, drained freely and incubated at approximately 50% of water holding capacity for ten months.

Times flooded	Species	Chao1	Simpson	Shannon	PD [a]
Archaea					
0	42 B [c]	90 A	0.87 B	3.96 B	6.4 B
1	79 A	183 A	0.96 A	5.60 A	11.4 A
2	75 AB	179 A	0.96 A	5.54 A	10.6 A
3	74 AB	180 A	0.96 A	5.51 A	11.4 A
6	83 A	270 A	0.97 A	5.75 A	11.9 A
7	85 A	191 A	0.97 A	5.91 A	12.7 A
9	83 A	186 A	0.98 A	5.91 A	12.3 A
10	81 A	219 A	0.96 A	5.67 A	12.5 A
MSD [b]	33	197	0.06	1.22	3.2
F value	4.26	1.55	8.24	6.46	10.07
P value	0.008	0.220	<0.001	0.001	<0.001
Bacteria					
0	951 A	1553 A	0.98 A	7.19 A	55.1 A
1	856 A	1315 AB	0.97 A	6.77 A	52.7 A
2	710 A	1061 B	0.98 A	6.90 A	47.8 A
3	729 A	1144 AB	0.98 A	6.94 A	46.5 A
6	843 A	1154 AB	0.97 A	6.87 A	57.8 A
7	852 A	1197 AB	0.98 A	7.33 A	59.9 A
9	631 A	956 B	0.99 A	7.47 A	48.2 A
10	689 A	1001 B	0.98 A	7.50 A	47.9 A
MSD	340	486	1.84	0.83	17.9
F value	2.42	3.71	0.149	2.94	1.99
P value	0.068	0.014	0.02	0.035	0.121

[a] PD: Phylogenetic diversity, [b] MSD: Minimum significant difference at the $P < 0.05$ (Proc GLM, Tukey, SAS Institute, 1989). [c] Values with the same capital letter (A,B) are similar in the flooded soils, i.e. within the column.
