# Supplementary material for: Reducing Salinity by Flooding an Extremely Alkaline and Saline Soil Changes the Bacterial Community but Its Effect on the Archaeal Community Is Limited
Source: Front Microbiol. 2017 Mar 27;8:466. doi: 10.3389/fmicb.2017.00466 (PMC5366314; doi:10.3389/fmicb.2017.00466)
Supplement: Supplementary file 11 [file Table4.PDF]

Supplementary Table S4. Bacterial groups affected significantly by flooding the soil monthly for 10 months.

Phylum	Class	Order	Family	Genus
Acidobacteria	Acidobacteria-6†	iii1-15†		
Actinobacteria*	Acidimicrobia†	Acidimicrobiales†	Microthrixaceae†	
	Actinobacteria	Actinomycetales	other Actinomycetales***	
			Dietziaceae***	*Dietzia****
			Micrococcaceae	*Nesterenkonia*†
			Nocardiaceae***	*Rhodococcus****
	Nitriliruptoria*	Nitriliruptorales†	Nitriliruptoraceae†	other Nitriliruptoraceae†, *Nitriliruptor**
Bacteroidetes*	Sphingobacteria†	Sphingobacteriales†		
	Cytophagia	Cytophagales	Cytophagaceae***	other Cytophagaceae*, *Sporocytophaga*†
Chloroflexi	TK17*	TK17**		
Firmicutes	Bacilli	Bacillales	Bacillaceae	other Bacillaceae**
	Bacilli	Lactobacillales†	Streptococcaceae†	*Streptococcus*†
	Clostridia	Clostridiales	Clostridiaceae	*Clostridium*†
		Natranaerobiales	ML1228J-1†	
Gemmatimonadetes	Gemm-5†			
Planctomycetes	Planctomycetia**	Pirellulales**	Pirellulaceae***	

Table S1. Continued.

Proteobacteria***	Alphaproteobacteria	other Alphaproteobacteria†		
		Caulobacterales	Caulobacteraceae	other Caulobacteraceae**, *Mycoplana*†
		Rhizobiales***	Beijerinckiaceae*	
			Brucellaceae***	*Ochrobactrum****
			Hyphomicrobiaceae†	*Devosia****
			Phyllobacteriaceae***	other Phyllobacteriaceae***, *Mesorhizobium***
		Sphingomonadales†	Sphingomonadaceae†	other Sphingomonadaceae†
	Betaproteobacteria	Burkholderiales	Comamonadaceae	other Comamonadaceae†, *Delfia*†, *Limnobacter*†, *Variovorax****
	Gammaproteobacteria***	other Gammaproteobacteria*		
		Alteromonadales	[Chromatiaceae] †	other [Chromatiaceae] †
		Chromatiales	other Chromatiales**	
			Chromatiaceae	other Chromatiaceae*
			Ectothiorhodospiraceae	other Ectothiorhodospiraceae*
		Oceanospirillales**	Halomonadaceae**	
		Pseudomonadales***	other Pseudomonadales***	
			Pseudomonadaceae***	other Pseudomonadaceae***, *Pseudomonas****
		Thiotrichales	Piscirickettsiaceae	other Piscirickettsiaceae†
		Xanthomonadales**	Xanthomonadaceae**	other Xanthomonadaceae*** *Stenotrophomonas**
Verrucomicrobia†	Opitutae†	other Opitutae*		
[Thermi]	Deinococci	Deinococcales	Trueperaceae	other Trueperaceae†
Unassigned**				

† Affected significantly at the P<0.05 level, * Affected significantly at the P<0.01 level, ** Affected significantly at the P<0.001 level, *** Affected significantly at the P<0.0001 level.
